# Identification of contributing genes of Huntington’s disease by machine learning

**DOI:** 10.1186/s12920-020-00822-w

**Published:** 2020-11-23

**Authors:** Jack Cheng, Hsin-Ping Liu, Wei-Yong Lin, Fuu-Jen Tsai

**Affiliations:** 1 Graduate Institute of Integrated Medicine, College of Chinese Medicine, China Medical University, Taichung, 40402 Taiwan; 2 Department of Medical Research, China Medical University Hospital, Taichung, 40447 Taiwan; 3 Graduate Institute of Acupuncture Science, College of Chinese Medicine, China Medical University, Taichung, 40402 Taiwan; 4 Brain Diseases Research Center, China Medical University, Taichung, 40402 Taiwan; 5 School of Chinese Medicine, China Medical University, Taichung, 40402 Taiwan; 6 Department of Biotechnology, Asia University, Taichung, 41354 Taiwan; 7 Children’s Medical Center, China Medical University Hospital, Taichung, 40447 Taiwan

**Keywords:** Huntington’s disease, Machine learning, Transcriptional regulation, Enrichment analysis

## Abstract

**Background:**

Huntington’s disease (HD) is an inherited disorder caused by the polyglutamine (poly-Q) mutations of the HTT gene results in neurodegeneration characterized by chorea, loss of coordination, cognitive decline. However, HD pathogenesis is still elusive. Despite the availability of a wide range of biological data, a comprehensive understanding of HD’s mechanism from machine learning is so far unrealized, majorly due to the lack of needed data density.

**Methods:**

To harness the knowledge of the HD pathogenesis from the expression profiles of postmortem prefrontal cortex samples of 157 HD and 157 controls, we used gene profiling ranking as the criteria to reduce the dimension to the order of magnitude of the sample size, followed by machine learning using the decision tree, rule induction, random forest, and generalized linear model.

**Results:**

These four Machine learning models identified 66 potential HD-contributing genes, with the cross-validated accuracy of 90.79 ± 4.57%, 89.49 ± 5.20%, 90.45 ± 4.24%, and 97.46 ± 3.26%, respectively. The identified genes enriched the gene ontology of transcriptional regulation, inflammatory response, neuron projection, and the cytoskeleton. Moreover, three genes in the cognitive, sensory, and perceptual systems were also identified.

**Conclusions:**

The mutant HTT may interfere with both the expression and transport of these identified genes to promote the HD pathogenesis.

## Background

Huntington’s disease (HD) is an inherited disorder that results in neurodegeneration characterized by chorea, loss of coordination, cognitive decline, depression, and psychosis [1]. The prevalence of HD is 13.7/100,000 in North America [2] and 16.8/100,000 for the elderly in Western Europe [3]. The neurodegeneration of HD is featured by a general shrinkage of the brain, especially the medium spiny neurons (MSNs) of the striatum [4]. The loss of cortical mass is an early hallmark in the pathology of HD [5].

HD is caused by the polyglutamine (poly-Q) mutations in the N-terminus of the HTT gene, which encodes huntingtin, a 350 kDa protein with ubiquitous expression [6]. The poly-Q extension is due to the abnormal CAG trinucleotide repeats in the mutant HTT (mHTT). The highest HTT expression level is observed in the neurons of the central nervous system with cytoplasmic-dominant localization and is associated with vesicle membranes [7]. Although HTT is known to be necessary for embryonic development and acts as a transcriptional regulator and protein scaffold in the synapse [8], the HD pathogenesis is still elusive [9]. To better understand the HD pathogenesis, we adopted machine learning (ML) on gene profiling dataset of the prefrontal cortex brain tissues of HD patients and controls and identified 66 disease-predicting genes. Their interaction network and potential roles in the HD pathogenesis are also discussed.

ML refers to computer algorithms that predict relying on the patterns of the data without using explicit instructions [10]. ML’s application on HD is focused on the diagnosis of HD from neuroimaging [11, 12]. Even though the emergence of a wide range of biological data of HD, including genomic profiling and electronic health records, a comprehensive understanding of the mechanism of HD from ML is so far unrealized, majorly due to the lack of needed data density [13]. For example, a previous ML study on RNA profiling of HD reported 4433 candidate genes from 16 samples [14], which is a typical high dimension, low sample size (HDLSS) situation, and ML may suffer from overfitting and low convergence. In this study, to harness the knowledge of the HD mechanism from the existing data, we tackled the data density issue by rationally reducing the dimension size, and identified the enriched pathways of HD by ML.

## Methods

### Data source

A gene profiling database of an essential sample size of HD and control is critical to this study. From the National Center for Biotechnology Information (NCBI) Gene Expression Omnibus (GEO), with the criteria “(“Huntington’s disease” AND “brain”) AND “Homo sapiens” [porgn:__txid9606]”, there were 342 series at the access date of June 18th, 2020. Out of them, there were four series with sample size > 100, including GSE72778, GSE33000, GSE25925, and GSE26927. We chose GSE33000 in this study since it provided the largest sample size of brain tissue profiling. The gene expression profile of the prefrontal cortex brain tissues of 157 HD patients and 157 non-demented control samples were retrieved from the GSE33000 dataset [15], which was profiled by microarray. This dataset contains 39,279 detected probes, of which 13,798 were annotated, and a total of 10,000 genes were profiled.

In solving equations, the number of parameters (in this case, age, sex, and gene profiles) should not exceed the number of equations (the sample size). Therefore, a preliminary screen of genes was essential. Since there were 10,000 genes profiled in GSE33000, the top 2.5% would yield approximately the gene numbers close to the total sample size 314. A criterion of fold change > 1.2 or < 0.85 resulted in 271 genes, which were selected along with HTT, as the input to build the prediction models. This fold-change criterion was chosen so that (1) the number of the selected genes was less than the number of total samples, and (2) the numbers of up-regulated and down-regulated genes were approximately equal (139 up and 132 down). Those genes with non-significant fold-change, i.e., *p* value of *T* test > 0.05 were neglected. After transposition (sample in the row and attributes in the column) and conversion of the disease status to binomials (1 = HD, 0 = control), the input dataset was constructed (Additional file 1: Table S1).

### Software and role assignment

**Fig. 1:**
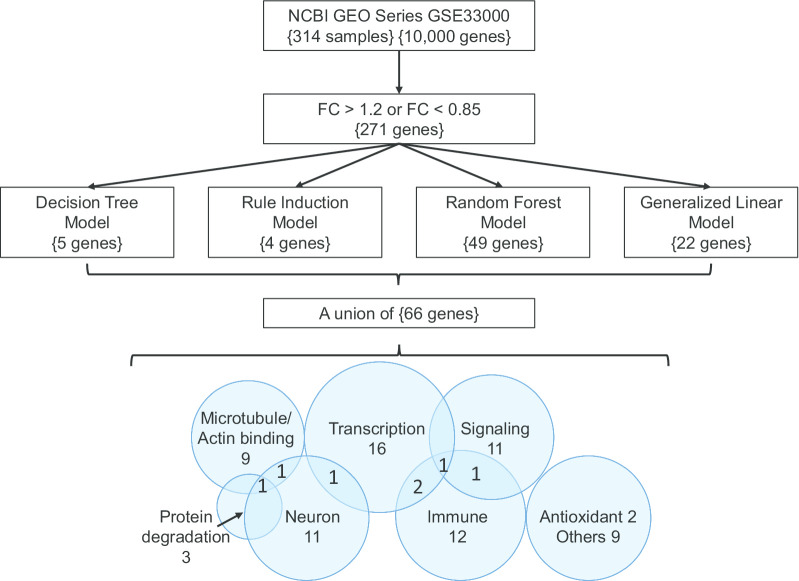
The study design and workflow. FC denotes the fold change of gene profiling. The curly brackets indicate the number of genes that passed the criteria or were identified in the data science models. The Venn diagram shows the number of genes in the enriched pathways

RapidMiner Studio version 9.5 (WIN64 platform) was registered to Jack Cheng and was executed under the Windows 10 operating system with Intel® Core™ i3-3220 CPU and 8 GB RAM. In addition to the age and sex of the samples, out of the 10,000 profiled genes, those expression fold change > 1.2 or < 0.85 of HD to control were assigned as the regular attributes (potential contributing factors to be analyzed in modeling operator) in the modeling. The disease status (1 = HD; 0 = CTRL) was assigned as the Label attribute (the predicted class in modeling operator). The sample ID was assigned as the ID attribute (not be used in modeling). Four models (decision tree, rule induction, random forest, and generalized linear model) of RapidMiner were used respectively with cross-validation to identify potential contributing genes of HD. The study design and over all workflow is shown in Fig. 1.

### Decision tree

**Fig. 2:**
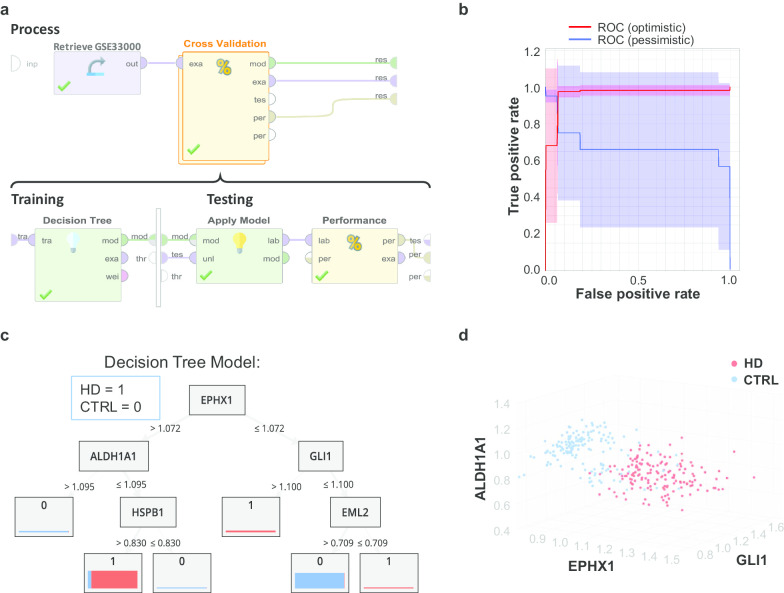
The decision tree model. **a** The program workflow. **b** The receiver operating characteristic (ROC) curve showing the performance of the prediction power of the model. **c** The modeled decision tree. A decision tree plots the “If… then” splitting of samples for prediction. The nodes denote the attributes, while the arrows denote the split, which meets a certain criterion. The number in the result box denotes the prediction result of the model (1 = HD; 0 = control), and the bar denotes the actual sample disease characteristic, bar sickness for sample size and bar segment for the proportion of HD samples (red = HD, blue = control). **d** The sample distribution in the 3-dimensional eigenspace of gene profiling. Red = HD, blue = control

A decision tree is a tree-like collection of nodes, representing a splitting rule for attributes to create a decision on the prediction class. The following parameters were used in RapidMiner modeling. Criterion: gain ratio; Maximal depth: 4; Prepruning and Pruning applied; Confidence: 0.01; Minimal gain: 0.01; Minimal leaf size: 2; Minimal size for a split: 4; Number of pre-pruning alternatives: 3. The program workflow is illustrated in Fig. 2a.

### Rule induction

The Rule Induction model develops a set of hypotheses that account for the most positive examples, but the least negative examples. The following parameters were used in RapidMiner modeling. Criterion: information gain; Sample ratio: 0.9; Pureness: 0.9; Minimal prune benefit: 0.25.

### Random forest

A random forest is an ensemble of random decision trees. The following parameters were used in RapidMiner modeling. The number of trees: 30; Criterion: gain ratio; Maximal depth: 4; Apply pruning with Confidence: 0.01; Apply pre-pruning with Minimal gain: 0.01; Minimal size for a split: 31 (~ 1/10 sample size); Minimal leaf size: 8; Number of pre-pruning: 3; Voting strategy: confidence vote.

### Generalized linear model

RapidMiner executes the GLM algorithm using H2O 3.8.2.6., which fits generalized linear models to the data by maximizing the log-likelihood and determines predictors with non-zero coefficients. These parameters were used in the modeling. Family: binomial; Solver: IRLSM; Use regularization; Do lambda search with the number of lamdas = 31 (~ 1/10 sample size) and early stopping of tolerance 0.01 after three rounds; Standardize and add interception.

### Cross-validation of models

In RapidMiner, the cross-validation has two subprocesses: a training subprocess and a testing subprocess. The training subprocess produces a trained model to be applied to the testing subprocess for the performance evaluation. In this study, the samples were randomly divided into ten subsets, with an equal number of samples. Each of the ten subsets was iterationaly used in the testing subprocess to evaluate the trained model from the other nine subsets. The performance of a model can be evaluated by its accuracy, precision, and recall, which are defined as below:

$$\begin{aligned} & {\text{Accuracy}}\, = \,\left( {{\text{TP}}\, + \,{\text{TN}}} \right)/({\text{TP}}\, + \,{\text{FP}}\, + \,{\text{FN}}\, + \,{\text{TN}}) \\ & {\text{Precision}}\, = \,{\text{TP}}/({\text{TP}}\, + \,{\text{FP}}) \\ & {\text{Recall}}\, = \,{\text{TP}}/({\text{TP}}\, + \,{\text{FN}}) \\ \end{aligned}$$

where T = true, F = false, P = positive, and N = negative.

A receiver operating characteristic (ROC) curve represents the sensitivity, or true positive rate, vs. false positive rate. It is calculated by first ordering the classified examples by confidence. Then all the examples are taken into account with decreasing confidence. The x-axis represents the false positive rate, and the y-axis represents the true positive rate. For optimistic (red) possibilities to calculate ROC curves, the correct classified examples are taken into account before looking at the false classifications, and the area in the red denotes the confidence interval. For pessimistic (blue) possibilities to calculate ROC curves, the wrong classifications are taken into account before looking at correct classifications, and the area in the blue denotes the confidence interval.

### Gene enrichment analysis and interaction network

For gene enrichment analysis, the identified gene symbols were used as the input to KOBAS 3.0 [16] (http://kobas.cbi.pku.edu.cn/kobas3/), utilizing the gene-list enrichment tool with default statistical criteria and specifying Homo sapiens species. For the gene interaction network, the identified gene symbols were used as the input to STRING: functional protein association networks [17] (https://string-db.org/).

## Results

### Decision tree identified EPHX1, ALDH1A1, and GLI1

A decision tree is a machine-learning algorithm to split rule for attributes (genes in this study) to create a decision on the prediction class (whether the sample is HD or not). A cross-validation strategy was used to train the model and to evaluate its performance (Fig. 2a). The machine-learned model is shown in Fig. 2c, which contains five genes, epoxide hydrolase 1 (EPHX1), aldehyde dehydrogenases 1 (ALDH1A1), zinc finger protein GLI1 (GLI1), heat shock protein beta-1 (HSPB1), and Echinoderm microtubule-associated protein-like 2 (EML2). These five genes served as part of the input for the enrichment and network analysis. The performance of this model is shown as a receiver operating characteristic (ROC) curve in Fig. 2b, with an accuracy of 90.79 ± 4.57%, a precision of 87.26 ± 6.95%, and a recall of 96.17 ± 3.30%. The separation of samples in the eigenspace of EPHX1, ALDH1A1, and GLI1 is shown in Fig. 2d. EPHX1 catalyzes epoxides and may play a role in the metabolism of epoxide-containing fatty acids [18]. ALDH1A1 may detoxify aldehydes in the brain [19]. GLI1 acts as a transcriptional activator, which regulates genes of neuroprotection [20]. HSPB1 is a molecular chaperone that maintains denatured proteins in a folding-competent state and exerts a cytoprotective effect by proteostasis [21]. EML2 is a tubulin-binding protein which inhibits microtubule nucleation and growth, and microtubules required for autophagy of aggregated huntingtin [22]. These identified genes participate in catalyzing ROS-producing chemicals, proteostasis, transcriptional regulation of neuroprotective genes. Altogether, the dysregulation of these genes may advance HD pathological progress.

### Rule induction identified EPHX1, OTP, and ITPKB

**Fig. 3:**
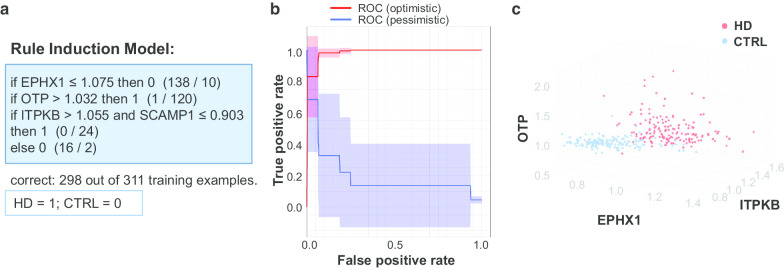
The rule induction model. **a** The receiver operating characteristic (ROC) curve showing the performance of the prediction power of the model. **b** The modeled rule induction. The number after “if … then” denotes the prediction result of the model (1 = HD; 0 = control), and the numbers in the parentheses (X/Y) denote the actual sample disease characteristic, X for the number of HD samples and Y for the number of control. **c** The sample distribution in the 3-dimensional eigenspace of gene profiling. Red = HD, blue = control

A rule induction model is a machine-learning algorithm, by judging the gene expression profiling in this study, that account for the most positive examples (HD), but the least negative examples (control). A cross-validation strategy was used to train the rule induction model and to evaluate its performance. The machine-learned model is shown in Fig. 3a, which contains four genes, EPHX1, homeobox protein orthopedia (OTP), inositol-trisphosphate 3-kinase B (ITPKB), and secretory carrier-associated membrane protein 1 (SCAMP1). These four genes also served as part of the input for the enrichment and network analysis. The performance of the rule induction model is shown as a ROC curve in Fig. 3b, with an accuracy of 89.49 ± 5.20%, a precision of 93.74 ± 6.81%, and a recall of 85.25 ± 11.10%. The separation of samples in the eigenspace of EPHX1, OTP, and ITPKB is shown in Fig. 3c. OTP is a homeobox protein with RNA polymerase II-specific DNA-binding transcription factor activity and may involve in the differentiation of hypothalamic neuroendocrine cells [23]. ITPKB is a kinase catalyzing inositol-trisphosphate 3 and may regulate neurite outgrowth by mediating MAPK cascade and RAS signal transduction [24]. SCAMP1 is a component of the recycling carrier that transports between endosomes, and Golgi complex, and the plasma membrane [25].

### Random forest identified 49 genes

**Fig. 4:**
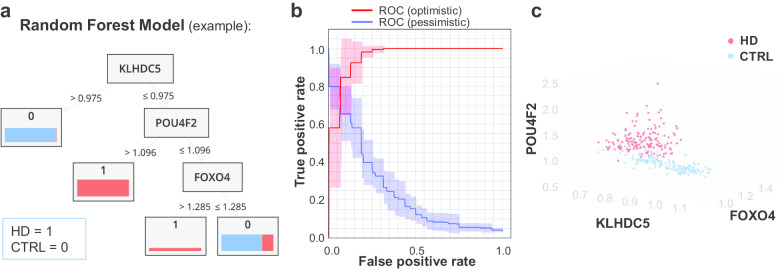
The random forest model. **a** The receiver operating characteristic (ROC) curve showing the performance of the prediction power of the model. **b** An example of a decision tree in the modeled random forest. A decision tree plots the “If… then” splitting of samples for prediction. The nodes denote the attributes, while the arrows denote the split, which meets a certain criterion. The number in the result box denotes the prediction result of the model (1 = HD; 0 = control), and the bar denotes the actual sample disease characteristic, bar sickness for sample size and bar segment for the proportion of HD samples (red = HD, blue = control). **c** The sample distribution in the 3-dimensional eigenspace of gene profiling. Red = HD, blue = control

A random forest model is a machine-learning algorithm of a collection of decision trees with voting hypotheses, by judging the gene expression profiling in this study, that account for the most positive examples (HD), but the least negative examples (control). A cross-validation strategy was used to train the random forest model and to evaluate its performance. The identified 30 decision trees and 49 non-redundant genes of the random forest are listed in Additional file 2: Table S2. These 49 genes served as part of the input for the enrichment and network analysis. One example of the machine-learned tree model is shown in Fig. 4a, which contains three genes, Kelch-like protein 42 (KLHDC5/KLHL42), POU domain class 4 transcription factor 2 (POU4F2), and forkhead box protein O4 (FOXO4). The performance of the random forest is shown as a ROC curve in Fig. 4b, with an accuracy of 90.45 ± 4.24%, a precision of 87.25 ± 4.72%, and a recall of 94.79 ± 6.10%. The separation of samples in the eigenspace of KLHDC5, POU4F2, and FOXO4 is shown in Fig. 4c. KLHDC5 is a component of the BTB-CUL3-RBX1 E3 ubiquitin-protein ligase complex, which mediates the ubiquitination of KATNA1 and regulates the microtubule dynamics in mitotic progression and cytokinesis [26]. POU4F2 is an RNA polymerase II specific transcription factor, which cooperates with TP53 to increase transcriptional activation of BAX promoter activity mediating neuronal cell apoptosis [27]. FOXO4 is a transcription factor, which regulates insulin signaling pathway, hypoxia-induced response, cell cycle, and proteasome activity [28].

### Generalized linear model identified 53 genes

**Fig. 5:**
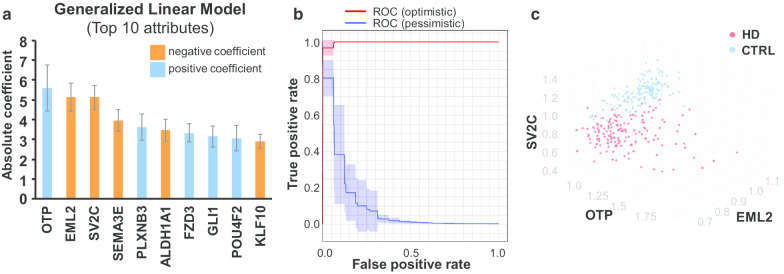
The generalized linear model. **a** The receiver operating characteristic (ROC) curve showing the performance of the prediction power of the model. **b** The top ten genes with the highest absolute coefficients in the model. The bar color denotes the polarity of the coefficient (orange = negative; blue = positive). **c** The sample distribution in the 3-dimensional eigenspace of gene profiling. Red = HD, blue = control

A generalized linear model (GLM) is a machine-learning algorithm that maximizes the log-likelihood (prediction power of whether a sample is an HD) and determines predictors (the gene profiling) with non-zero coefficients indicating a linear contribution of the gene profiling to the prediction. A cross-validation strategy was used to train the GLM and to evaluate its performance. The coefficients of the input genes are listed in Additional file 3: Table S3. There are 53 genes with a non-zero coefficient. We further selected more contributive genes by setting a threshold of the absolute value of the coefficient greater than 1. These 22 genes are also listed in Additional file 3: Table S3, and served as part of the input for the enrichment and network analysis. The top ten genes of coefficients are shown in Fig. 5a. The performance of the GLM is shown as a ROC curve in Fig. 5b, with an accuracy of 97.46 ± 3.26%, a precision of 95.96 ± 5.14%, and a recall of 99.38 ± 1.98%. The separation of samples in the eigenspace of gene profiling of the top 3 genes, OTP, EML2, and synaptic vesicle glycoprotein 2C (SV2C), is shown in Fig. 5c. SV2C regulates secretion in neural cells by enhancing selectively low-frequency neurotransmission [29].

### Gene enrichment and interaction network analysis

**Fig. 6:**
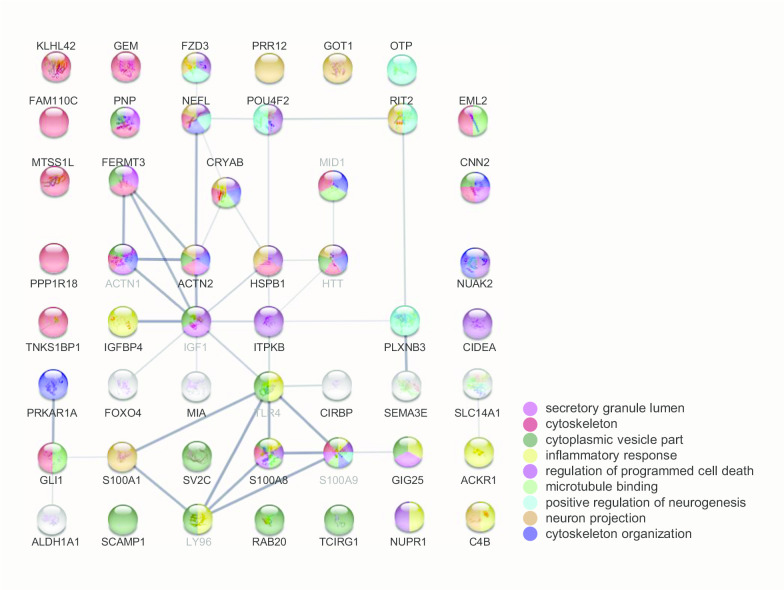
Gene ontology and interaction network. The enriched gene ontology is represented by different colors of the nodes. The strength of the evidence of the interaction is represented by the darkness of the edges. Genes identified in this study are labeled with black font. Genes manually added are labeled with grey font

The union of the identified 66 non-redundant genes from machine learning is summarized in Additional file 4: Table S4, and served as the input for the enrichment and network analysis. The significant enrichment in Gene Ontology, KEGG disease/ NHGRI GWAS catalog, and KEGG pathway are listed in Additional file 5, 6, 7: Tables S5, S6, and S7, respectively. As summarized in the lower part of Fig. 1, the enriched characteristics of the genes are transcription (16 genes), immune (12), neuron (11), signaling (11), and microtubule/actin binding. While Fig. 6 shows the interaction network, which indicates HSPB1, ITPKB, CRYAB, ACTN2, FERMT3, NEFL, POU4F2, RIT2, and PLXNB3 are closely related to HTT and may serve as pivotal points exerting consequences of HTT-polyQ mutation in HD. CRYAB is a chaperone preventing aggregation of proteins under stress conditions [30]. ACTN2 is an F-actin cross-linking protein that participates in cell adhesion, MAPK cascade, apoptosis, and the regulation of NMDA receptor activity [31]. FERMT3 is an integrin-binding protein that plays a part in cell adhesion and activation of the integrin-mediated signaling pathway [32]. NEFL is an intermediate filament protein that maintains neuronal caliber essential for sensorimotor function and spatial orientation [33]. RIT2 is a small GDP-binding protein which acts as molecular switches for intracellular signaling cascades in neuron and is regulated by POU4 transcription factors [34]. PLXNB3 is a SEMA receptor regulating cell adhesion, chemotaxis, and neuron projection [35]. Noticeably, HSPB1, ITPKB, and POU4F2 are also key attributes in the machine-learning models.

## Discussion

In this study, from the profiling of 157 HD and 157 controls, we identified 66 potential contributing genes of HD using machine learning models of the decision tree, rule induction, random forest, and generalized linear model. The identified genes enriched the gene ontology of transcriptional regulation, inflammatory response, neuron projection, and cytoskeleton (Fig. 6). These pathways are connected by hubs of microtubule/actin binding, which may imply that mutant HTT mediates the HD pathological progress through these pathways via its interaction with the cytoskeleton or via transcriptional regulation capacity. We will discuss the enriched biological functions and the relevant genes in HD pathogenesis.

C20orf54 (SLC52A3) encodes a plasma membrane transporter mediating the uptake of vitamin B2/riboflavin that is vital in biochemical oxidation–reduction reactions [36]. The mutation of SLC52A3 may cause degenerative disorders like Brown-Vialetto-Van-Laere syndrome (BVVL) [37] and Amyotrophic lateral sclerosis (ALS) [38]. Although the role of oxidative damage in HD pathogenesis has been discussed for decades [39]. The potential role of SLC52A3 in the riboflavin-related oxidative damage in HD has not been noticed yet. The other two genes with detoxifying ability identified in this study are ALDH1A1 and MT1H, which detoxifies aldehydes [19] or copper ions [40], respectively. Whether aldehydes or copper ion detoxification participates in HD pathogenesis requires further study.

One of the hallmark pathological features of HD is the intracellular aggregates of mutant HTT, termed inclusion bodies (IBs). The insufficient clearance of toxic forms of mutant HTT is postulated as one hypothesis of HD pathogenesis [41]. Three genes involving in protein degradation were identified in this study: CRYAB, HSPB1, and KLHDC5. Expression of CRYAB influences autophagy and protein aggregation [42]. HSPB1 mutation may impair autophagy and cause neuropathy [43]. KLHDC5 is an adapter of the BTB-CUL3-RBX1 E3 ubiquitin-protein ligase and regulates the ubiquitin–proteasome system [44]. Currently, there is a lack of knowledge of the roles played by CRYAB, HSPB1, and KLHDC5 in HD pathogenesis.

Although it is unclear whether neuroinflammation has an active influence or is a reactive process during the HD pathogenesis, both innate and adaptive immune systems may play important roles in HD [45]. The former includes activation of microglia, increased proinflammatory cytokines, impaired translocation of macrophages, and complement factors. The later includes T-cell priming by dendritic cells (DCs). In this study, the identified innate immunity genes include C4B, DARC, RAB20, SBNO2, SCAMP1, SERPINA3, and S100A8, while the adaptive immunity genes include PNP, TCIRG1, and TMEM176A. More specifically, C4B is one complement factor [46]; DARC is a chemokine receptor [47], RAB20 involves in endocytosis [48]; SBNO2 regulates the transcription of NF-κB in macrophages [49]; SCAMP1 regulates the neutrophil degranulation [50]; SERPINA3 inhibits neutrophil cathepsin G and mast cell chymase [51]. S100A8 induces neutrophil chemotaxis and therefore participates in both innate and adaptive immune systems [52], while PNP regulates T cell proliferation [53]; TCIRG1 isoform b is an inhibitory receptor on T cells [54]; TMEM176A regulates the dendritic cell differentiation [55].

Since the discovery of the involvement of HTT in the transcription regulation of P53 and CREB [56], dysregulation of transcription by mHTT becomes a popular hypothesis of HD pathogenesis [9]. In this study, we identified several transcription regulatory genes, including CIDEA, CIRBP, FOXO4, GLI1, KLF10, NUPR1, OTP, POU4F2, PRKAR1A, RIT2, SFRS5, TBX15, and TEAD2. Notably, OTP, RIT2, and POU4F2 also regulate neurogenesis (Fig. 6), while CIRBP, FOXO4, GLI1, and NUPR1 regulate gene expression under stress circumstances [57–60]. Furthermore, CIDEA, KLF10, PRKAR1A, TBX15, and TEAD2 regulate gene expression of apoptosis control [61–65]. Whether these genes are driving forces or merely passengers in HD pathogenesis requires further investigation.

Wild-type HTT is a scaffolding protein interacting with β-tubulin and microtubules [66]. It also interacts with the dynactin complex and regulates intracellular trafficking processes [67]. In this study, we identified several microtubule/actin binding genes, including ABBA-1, ACTN2, CNN2, FAM110C, KIAA1949, and SEMA3E. Likewise, dysregulation of these genes may disturb intracellular trafficking processes with mHTT.

Wild-type HTT also plays a critical role at the synapse. It is associated with the synaptic vesicles at the pre-synapse [7] and is associated with the scaffolding protein PSD95 at the postsynaptic density [68]. Moreover, HTT is required during the formation of cortical and striatal excitatory synapses [69]. However, the role of HTT in the neuron is still obscure. In this study, we identified several neuronal genes, including GOT1, HTR2C, PLXNB3, and SV2C. GOT1 synthesizes and regulates the quantity of glutamate [70], which is a key neurotransmitter. Besides, HTR2C is a serotonin receptor mediating excitatory neurotransmission [71], while PLXNB3 is a SEMA5A receptor mediating axon guidance [72]. Moreover, SV2C is a synaptic vesicle glycoprotein mediating low-frequency neurotransmission [29]. The dysregulation of these genes may provoke HD symptoms.

We also identified three genes in the cognitive, sensory, and perceptual systems: DOPEY2, EML2, and NEFL. The deficits in these domains are the hallmark symptoms in HD and may serve as diagnostic cues [73–75]. The overexpression of DOPEY2 may contribute to mental retardation [76], while EML2 has a role in visual perception [77]. Moreover, mutations in NEFL cause inherited motor and sensory neuropathy [78]. Although thousands of paper report HD and sensorimotor dysfunction, no one notices their potential roles in HD pathological symptoms, especially sensorimotor dysfunction. In this study, we revealed that HTT mutation might exert pathological interference on NEFL by two independent routes, as shown in Fig. 6. One route is by dysregulation of transcription through POU4F2. The other route is by dysregulation of the cytoskeleton through ACTN2 and CRYAB.

Finally, we compared our results with the existing ML-based method [14] for identifying HD-contributing genes and checked whether these 66 contributing genes are included in the known HD gene set. Out of the 66 genes, 13 genes are mutually identified in both ML studies. Furthermore, 21 of the 66 genes have been identified in previous HD studies. This information was provided in Additional file 8: Table S8.

## Conclusions

Machine learning using the decision tree, rule induction, random forest, and generalized linear model identified 66 potential contributing genes of HD from the expression profiles of postmortem prefrontal cortex samples of 157 HD and 157 controls. These genes participate in oxidation–reduction reactions, protein degradation, immunity, transcription, neural transduction, and perception. The mHTT may interfere with both the expression and transport of these genes to promote the HD pathogenesis.

## Supplementary information

**Additional file 1: Table S1.** The input file to RapidMiner program of this study. Columns are attributes, while rows are samples. The column “HD” is a binominal attribute, i.e., 1 or 0, describing whether the sample is diagnosed with HD or not, respectively.

**Additional file 2: Table S2.** The identified 30 decision trees and 49 non-redundant genes of the random forest.

**Additional file 3: Table S3.** The coefficients of the generalized linear model.

**Additional file 4: Table S4.** The list of the identified genes of this study. A tick sign or blank denotes whether this gene was identified in the corresponding algorithm or not, respectively.

**Additional file 5: Table S5.** The enriched Gene Ontology of the identified genes.

**Additional file 6: Table S6.** The enriched KEGG DISEASE, NHGRI GWAS Catalog, and OMIM of the identified genes.

**Additional file 7: Table S7.** The enriched KEGG PATHWAY, Reactome, and PANTHER database of the identified genes.

**Additional file 8: Table S8.** The comparison with the existing ML-based HD study, and whether these 66 genes are included in the known HD studies.

## Data Availability

The dataset supporting this article’s conclusions is available in the NCBI GEO repository, with accession number GSE33000 in https://www.ncbi.nlm.nih.gov/geo/query/acc.cgi?acc=GSE33000. The data supporting the conclusions of this article are included within the article and its additional files. The machine learning platform RapidMiner Studio is available at https://rapidminer.com/.

## Abbreviations

HD: Huntington’s disease
poly-Q: Polyglutamine
mHTT: Mutant HTT
ML: Machine learning
GLM: Generalized linear model
ROC: Receiver operating characteristic
ALS: Amyotrophic lateral sclerosis
TP: True positive
FP: False positive
TN: True negative
FN: False negative
